# Novel enzyme-based reduced representation method for DNA methylation profiling with low inputs

**DOI:** 10.1093/nar/gkaf558

**Published:** 2025-06-30

**Authors:** Qianli Liu, Kathryn A Helmin, Zachary D Dortzbach, Carla P Reyes Flores, Manuel A Torres Acosta, Jonathan K Gurkan, Anthony M Joudi, Nurbek Mambetsariev, Luisa Morales-Nebreda, Mengjia Kang, Luke Rasmussen, Xóchitl G Pérez-Leonor, Hiam Abdala-Valencia, Benjamin D Singer

**Affiliations:** Division of Pulmonary and Critical Care Medicine, Northwestern University Feinberg School of Medicine, Chicago, IL 60611, United States; Driskill Graduate Program, Northwestern University Feinberg School of Medicine, Chicago, IL 60611, United States; Division of Pulmonary and Critical Care Medicine, Northwestern University Feinberg School of Medicine, Chicago, IL 60611, United States; Division of Pulmonary and Critical Care Medicine, Northwestern University Feinberg School of Medicine, Chicago, IL 60611, United States; Division of Pulmonary and Critical Care Medicine, Northwestern University Feinberg School of Medicine, Chicago, IL 60611, United States; Driskill Graduate Program, Northwestern University Feinberg School of Medicine, Chicago, IL 60611, United States; Division of Pulmonary and Critical Care Medicine, Northwestern University Feinberg School of Medicine, Chicago, IL 60611, United States; Medical Scientist Training Program, Northwestern University Feinberg School of Medicine, Chicago, IL 60611, United States; Division of Pulmonary and Critical Care Medicine, Northwestern University Feinberg School of Medicine, Chicago, IL 60611, United States; Driskill Graduate Program, Northwestern University Feinberg School of Medicine, Chicago, IL 60611, United States; Medical Scientist Training Program, Northwestern University Feinberg School of Medicine, Chicago, IL 60611, United States; Division of Pulmonary and Critical Care Medicine, Northwestern University Feinberg School of Medicine, Chicago, IL 60611, United States; Division of Allergy and Immunology, Northwestern University Feinberg School of Medicine, Chicago, IL 60611, United States; Division of Pulmonary and Critical Care Medicine, Northwestern University Feinberg School of Medicine, Chicago, IL 60611, United States; Division of Pulmonary and Critical Care Medicine, Northwestern University Feinberg School of Medicine, Chicago, IL 60611, United States; Division of Health and Biomedical Informatics, Department of Preventive Medicine, Northwestern University Feinberg School of Medicine, Chicago, IL 60611, United States; Division of Pulmonary and Critical Care Medicine, Northwestern University Feinberg School of Medicine, Chicago, IL 60611, United States; Division of Pulmonary and Critical Care Medicine, Northwestern University Feinberg School of Medicine, Chicago, IL 60611, United States; Division of Pulmonary and Critical Care Medicine, Northwestern University Feinberg School of Medicine, Chicago, IL 60611, United States; Department of Biochemistry and Molecular Genetics, Northwestern University Feinberg School of Medicine, Chicago, IL 60611, United States; Simpson Querrey Institute for Epigenetics, Northwestern University Feinberg School of Medicine, Chicago, IL 60611, United States; Simpson Querrey Lung Institute for Translational Science (SQ LIFTS), Northwestern University Feinberg School of Medicine, Chicago, IL 60611, United States

## Abstract

Commonly used bisulfite-based procedures for DNA methylation sequencing can degrade DNA, worsening signal-to-noise ratios in samples with low DNA input. Enzymatic methylation sequencing (EM-seq) has been proposed as a less biased alternative for methylation profiling with greater genome coverage. Reduced representation approaches enrich samples for CpG-rich genomic regions, thereby enhancing throughput and cost effectiveness. We hypothesized that enzyme-based technology could be adapted for reduced representation methylation sequencing to enable DNA methylation profiling of low-input samples. We leveraged the well-established differences in methylation profile between mouse CD4^+^ T cell populations to compare the performance of our reduced representation EM-seq (RREM-seq) procedure against an established reduced representation bisulfite sequencing (RRBS) protocol. While the RRBS method failed to generate reliable DNA libraries when using <2 ng of DNA, the RREM-seq method successfully generated reliable DNA libraries from 1–25 ng of mouse and human DNA. Low-input (≤2-ng) RREM-seq libraries demonstrated superior regulatory genomic element coverage compared with RRBS libraries with >10-fold higher DNA input. RREM-seq also successfully detected lineage-defining methylation differences between alveolar conventional T and regulatory T cells obtained from patients with severe SARS-CoV-2 pneumonia. Our RREM-seq method enables single-nucleotide resolution methylation profiling using low-input samples, including from clinical sources.

## Graphical Abstract

**Graphical Abstract:**
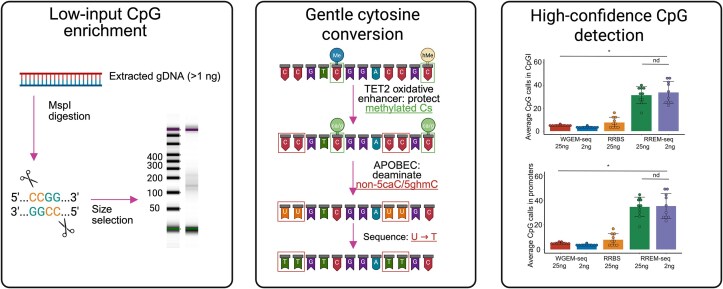

## Introduction

In mammalian genomes, 60%–80% of cytosine-phospho-guanine (CpG) dinucleotides are modified with a methyl group at the fifth carbon position (5mC) [1]. These CpG dinucleotide residues are nonuniformly distributed throughout the genome, and are clustered in GC-rich CpG islands (CpGIs) [2]. Within regulatory genomic elements, e.g. CpGIs and gene promoter regions, methylated CpGs tend to be associated with transcriptional repression [3]. Currently, bisulfite-based methods, including whole-genome bisulfite sequencing (WGBS) and reduced representation bisulfite sequencing (RRBS), are considered the gold standard for methylation mapping with single-nucleotide resolution [4].

Despite its popularity, bisulfite sequencing requires extreme temperature and pH during library preparation, which cause DNA degradation, especially among unmethylated cytosines [5]. This disproportionate damage of unmethylated cytosines results in libraries with inaccurate GC content representation [6]. Enzymatic cytosine conversion has been proposed as an alternative method for methylation mapping. Compared with bisulfite sequencing, enzymatic methylation sequencing (EM-seq) has more balanced genome coverage with less GC bias and identifies more genomic features with the same number of reads, boosting signal-to-noise ratios in low-input samples [5].

Compared with whole-genome methylation sequencing, reduced representation methylation sequencing enriches DNA samples for CpG-rich genomic regions via restriction enzyme (e.g. MspI) digestion. This enrichment step prior to cytosine conversion greatly enhances sample throughput and reduces sequencing costs, allowing sample multiplexing and profiling of a larger sample size to enhance statistical power at a given number of biological replicates [7, 8]. A previous study by Vaisvila *et al.* [5] demonstrated EM-seq’s superior performance in whole-genome methylation sequencing compared with WGBS, yet whether the enzymatic cytosine conversion technology could be adapted for reduced representation methylation sequencing remains unknown.

In this study, we performed methylation profiling of mouse splenic T cell subsets and alveolar T cell subsets from patients with severe SARS-CoV-2 pneumonia to compare the performance of bisulfite-based and EM-seq methods, including a novel reduced representation EM-seq (RREM-seq) approach. We chose specific T cell subsets—CD4^+^ conventional T (Tconv) cells and FOXP3^+^ regulatory T (Treg) cells—that we and others demonstrated to have distinct methylation profiles in mice and humans [9–12]. Specifically, Treg cells are hypomethylated compared with CD4^+^ conventional T cells within defined Treg-specific super-enhancer (Treg-SE) regions, which are lineage-specifying elements that determine Treg cell identify and function in the lung and other organs [9]. We found that RREM-seq is a reliable method to perform low-input methylation profiling and demonstrated a superior signal-to-noise ratio compared with RRBS in low-input samples.

## Materials and methods

### Ethics statement

All mouse procedures were approved by the Northwestern University IACUC under protocols IS00012519 and IS00017837. All patients were enrolled in the Successful Clinical Response in Pneumonia Therapy (SCRIPT) study under Northwestern University IRB STU00204868, providing informed consent in accord with the Declaration of Helsinki.

### Mouse tissue preparation and flow cytometry sorting

Spleens were harvested from healthy *Foxp3^YFP-Cre^* mice (Jackson Labs strain #016959), in which yellow fluorescent protein (YFP) marks the Treg cell lineage. Single-cell suspensions were prepared and red blood cells were removed via ACK lysis buffer (Thermo Fisher). Staining was performed using reagents listed in Supplementary Table S1. Cell sorting of conventional T (CD3ϵ^+^ CD4^+^ *Foxp3*-YFP^−^) and Treg (CD3ϵ^+^ CD4^+^ CD25^+^ *Foxp3*-YFP^+^) cells was performed using BD Biosciences FACSAria SORP instruments with FACSDiva software and Miltenyi Biotec MACSQuant Tyto instruments.

### T cell samples from patients with severe SARS-CoV-2 pneumonia

Bronchoalveolar lavage (BAL) samples were collected from mechanically ventilated patients with severe SARS-CoV-2 pneumonia within 48 h of intubation. For SCRIPT participants, the first BAL (study day 0) is performed within 48 h following intubation. The etiology of pneumonia was determined based on clinical and BAL fluid analysis by consensus of five pulmonary and critical care medicine physicians [13]. All BAL samples were sorted and analyzed using our published flow cytometry methods [9], and then stored at −80°C. In this study, we included the sorted day 0 alveolar Treg cells (live FSClo SSClo CD3ϵ^+^ CD4^+^ CD25hi CD127lo) and Tconv cells (live FSClo SSClo CD3ϵ^+^ CD4^+^ cells not in the Treg cell gate) [9] from the SCRIPT study and performed methylation profiling using RREM-seq.

### Methylation library generation

Flow cytometry-sorted cells were first lysed with QIAGEN RLT Plus and then genomic DNA was extracted using the AllPrep DNA/RNA Micro Kit (QIAGEN). For WGEM-seq libraries, 25 ng of genomic DNA was first fragmented to a size of 240–290 bp using a Covaris E220 sonicator and then enzymatically converted with TET2 and APOBEC (New England Biolabs) per the manufacturer’s instructions. For WGBS libraries, 25 ng of genomic DNA was bisulfite converted with the EZ DNA Methylation-Lightning Kit (Zymo Research) per the manufacturer’s protocol. A restriction enzyme digestion step was included for RRBS and RREM-seq libraries, in which genomic DNA was fragmented with MspI (New England Biolabs) and then size-selected for 100–250-bp fragments using solid-phase reversible immobilization beads (MagBio Genomics) as we described in previous publications [9, 14]. CpG-enriched genomic DNA was then bisulfite converted for RRBS libraries and enzymatically converted for RREM-seq libraries similar to the whole-genome libraries.

Random priming, adapter ligation, PCR (polymerase chain reaction) product clean-up, and final library amplification were performed using the NEBNext Enzymatic Methyl-seq Kit (New England BioLabs) for WGEM-seq libraries, and the Pico Methyl-Seq Library Prep Kit (Zymo Research) was used for WGBS, RRBS, and RREM-seq libraries. Bisulfite-based methods and enzyme-based methods required 10 and 8 PCR cycles for final amplification, respectively. Final library size distribution and quality were assessed via high-sensitivity screen tape (TapeStation 4200, Agilent). Unmethylated λ-bacteriophage DNA (1:200 mass ratio; New England Biolabs) was included in all samples to calculate unmethylated cytosine conversion efficiency. Whole-genome libraries were sequenced using 100 base-pair paired-end reads (NextSeq 2000 P3 reagent kit 200 Cycles; Illumina) with an average read count of 307 million reads per sample, and reduced representation libraries were sequenced using 75 base-pair single-end reads (NextSeq 2000 P2 reagent kit 100 Cycles; Illumina) with an average read count of 115 million reads per sample. Four samples were pooled for each run, and the pooled DNA methylation libraries were diluted to 2 nM for sequencing.

### Methylation library analysis

Methylation sequencing analysis was performed using our published Bismark-based pipelines [7, 9, 12]. After sequencing, raw binary base call (BCL) files were converted to FASTQ files using bcl-Convert (version 3.10.5; Illumina) and then trimming was performed using Trim Galore! (version 0.4.3). Bismark (version 0.21.0) was used to perform alignment to the reference genomes (mm10/GRCm38 assembly for mouse samples and hg38/GRCh38 assembly for patient samples) and methylation extraction. WGBS analysis was performed according to the Bismark instructions for pair-end post-bisulfite adapter tagging (PBAT) libraries [15]. Bismark coverage files were used for quantification using SeqMonk (version 1.48.0), R package DSS (version 2.46.0), and R package methylKit (version 1.24.0). Within-group and between-group Pearson correlation analysis of covered CpGs were performed using Methylkit (min.cov = 10). Pairwise methylation concordance analysis was performed using the united MethylKit dataset. For each CpG site, a concordant call between two samples was defined as having an absolute difference in β values of <0.15.

Bismark coverage files were imported into SeqMonk using the “Import Data” and “Bismark (Cov)” options. For CpG coverage analysis, SeqMonk probes were generated using the “Read Position Probe Generator” option. CpGs with at least 1, 5, and 10 reads were included in the 1×, 5×, and 10× minimum threshold analysis, respectively. For genomic feature coverage analysis, SeqMonk probes were generated using the “Feature Probe Generator” option. CpGI, transcription start site, and transcription end site coordinates were obtained from the SeqMonk GRCm38/GRCh38 annotation sets. All CpGI analysis included the surrounding CpGI shores. CpGI shores were defined as 2-kb regions flanking CpGIs, and promoters were defined as 1-kb regions flanking transcription start sites. For CpG coverage analysis, probes were quantified using the “Bisulphite methylation over features” pipeline. For CpG call analysis, probes were quantified using the “Read Count Quantification” function without correction and log transformation. Since Bismark coverage files consider a given CpG residue on different DNA strands as two independent positions, we estimated unique CpG coverage by only counting adjacent CpG calls once.

### Treg-SE

Published coordinates of Treg-SE elements [10] were first mapped from the original mm9 to the mm10 reference genome and then mapped to the hg38 reference genome using the UCSC Batch Coordinate Conversion (liftOver) tool with the default settings (minimum ratio of bases that must remap = 0.1).

### Statistics

Statistical analyses were performed in R (version 4.2.3), GraphPad Prism v10.3.0, and SeqMonk (version 1.48.0). All computational processing and analysis were performed using Northwestern University’s High-Performance Computing Cluster. A *P-*value or false discovery rate (FDR) *q* value <0.05 was considered statistically significant.

## Results

**Figure 1.**
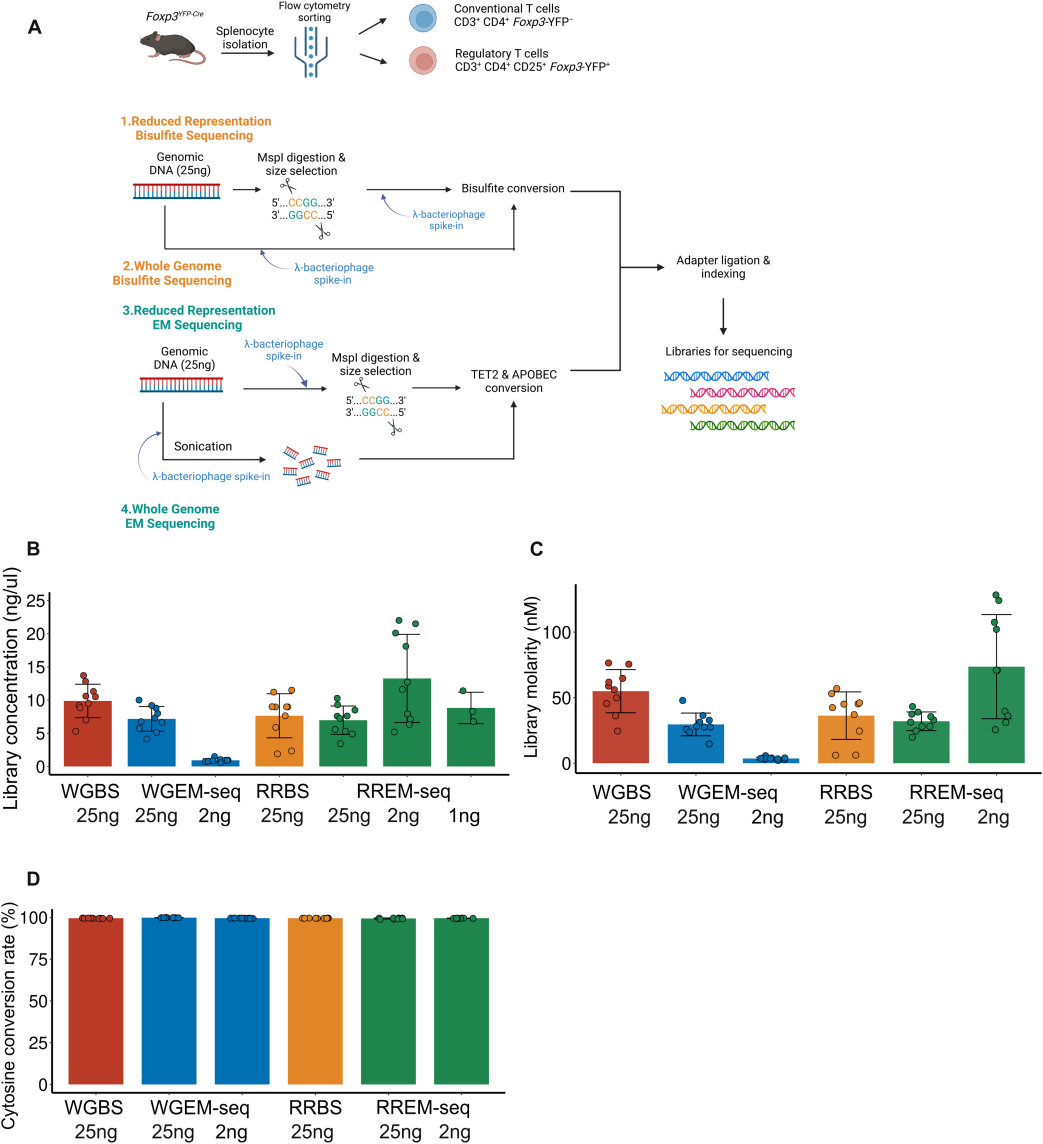
Construction and metrics of DNA methylation libraries. (**A**) Schematic representation of mouse splenic T cell sorting and DNA methylation library construction (created using biorender.com: https://BioRender.com/gaxr166). The CpG enrichment part is redrawn from Fig. 2E in [7]. DNA methylation library final concentration (**B**), molarity (**C**), and cytosine conversion rate (**D**) between four different methylation sequencing methods. Each data point represents one sample, and the bars represent average values of five biological replicates (five Tconv cell samples and five Treg cell samples). Summary plots show all data points with mean; error bars show standard deviation.

To compare the performance of bisulfite conversion and enzymatic conversion in 5mC detection (hereafter referred to as CpG detection), we performed WGBS and WGEM-seq, and RRBS and RREM-seq, leveraging mouse CD4^+^ Tconv and Treg cells as cell types with known differences in DNA methylation patterns at defined genomic elements. We used our previously published flow cytometry methods [11, 12, 16] to sort live Tconv and Treg cells from the spleens of *Foxp3^YFP-Cre^* mice, in which YFP marks the Treg cell lineage (Fig. 1A). We defined standard DNA input as >10 ng, low DNA input as 2–10 ng, and ultra-low DNA input as <2 ng. At low and ultra-low input levels, RREM-seq successfully generated acceptable quality libraries without the need for additional PCR amplification cycles (Supplementary Fig. S1A). These libraries fell within the expected size range, and primer contamination was not observed. While RRBS failed to generate reliable DNA libraries when using ≤2-ng inputs, the molarity, concentration, and quality of the low-input RREM-seq libraries was comparable to those of RRBS libraries with >10-fold higher DNA input (Fig. 1B and C, and Supplementary Fig. S1B). Unmethylated λ-bacteriophage DNA was included in all samples as an internal negative control, with an average assayed CpG methylation rate of 0.45%, indicating a >99% unmethylated cytosine conversion rate in all libraries (Fig. 1D).

**Figure 2.**
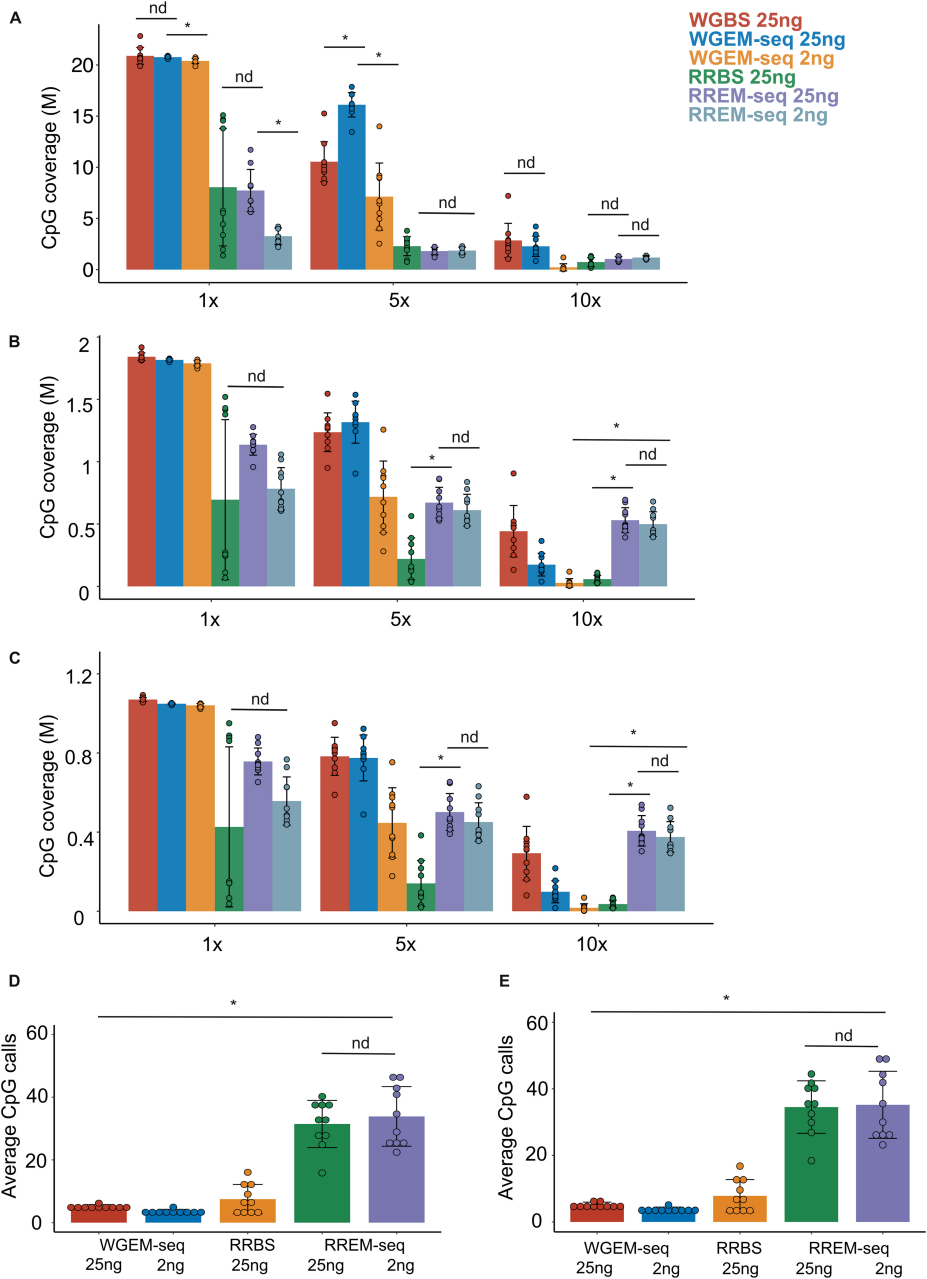
Global CpG coverage and CpG coverage at regulatory genomic elements. Global CpG detection (**A**), CpG detection within CpGIs and the surrounding CpGI shores (**B**), and CpG detection within promoters (**C**) at 1×, 5×, and 10× minimum thresholds (CpGs with at least 1, 5, and 10 reads were included in each analysis). Separate Kruskal–Wallis tests with two-stage linear step-up procedure of Benjamini, Krieger, and Yekutieli were performed for whole-genome methods (WGBS, standard-input WGEM-seq, and low-input WGEM-seq) and reduced representation methods (RRBS, standard-input RREM-seq, and low-input RREM-seq) at each minimum coverage threshold. Average number of CpG calls (read count of a given CpG) within CpGIs, including the surrounding CpGI shores (**D**) and promoters (**E**) at 1× minimum coverage. Analysis only includes CpGs with non-zero counts in each sample. Each data point represents one sample, and the bars represent average values of five biological replicates (five Tconv cell samples and five Treg cell samples). Error bars show standard deviation. *q < 0.05; nd, not a discovery, according to one-way ANOVA (Kruskal–Wallis test) using the two-stage linear step-up procedure of Benjamini, Krieger, and Yekutieli with *Q* = 5%.

At a minimum coverage depth of 1×, standard-input WGEM-seq and standard-input WGBS detected a similar number of CpG on average, while average global CpG coverage for low-input WGEM-seq is significantly lower (Fig. 2A). With increase of the coverage threshold to 5×, standard-input WGEM-seq detected significantly more CpGs than standard-input WGBS and low-input WGEM-seq. At 10× coverage depth, the differences in CpG detection between standard-input WGBS and standard-input WGEM-seq were negligible. Compared with RREM-seq, standard-input RRBS demonstrated a high degree of variation in CpG detection, particularly at 1× coverage depth. This variation in global CpG detection was highly correlated with RRBS library duplication rate (Supplementary Fig. S2A; *r*= −0.99; *P* < .001), suggesting that bisulfite-based cytosine conversion generates libraries with a more varied duplication rate compared with enzymatic cytosine conversion. At 1×, 5×, and 10× coverage thresholds, standard-input RRBS and standard-input RREM-seq detected a similar number of CpG on average (Fig. 2A). As inputs decreased from 25 to 2 ng, RREM-seq demonstrated a consistent CpG detection at 5× and 10× coverage thresholds, indicating that the performance of RREM-seq in high-confidence CpG detection is not correlated with input concentration at these input levels. While within-group correlation and concordance rate varied between RRBS replicates at 10× minimum coverage, standard-input and low-input RREM-seq replicates showed within-group correlation and concordance rate comparable to WGBS and WGEM-seq replicates (Supplementary Fig. S3A–F and J–O). Overlapping CpGs between WGBS and RREM-seq also demonstrated a higher and more consistent correlation and concordance compared with RRBS (Supplementary Fig. S3G–I and P–R). These results further support RREM-seq’s superior performance in accuracy and consistency when using various input levels.

We also compared CpG detection within CpG-rich regulatory genomic elements, including CpGIs (including the surrounding CpGI shores) and gene promoters between the four methylation sequencing methods. While RRBS and RREM-seq showed similar CpG detection within CpGIs and promoters at 1× coverage depth, standard-input and low-input RREM-seq detected significantly more CpGs within these functional genomic elements when increasing the coverage threshold to 5× and 10× (Fig. 2B and C). As inputs decreased from 25 to 2 ng, RREM-seq did not demonstrate a significant decrease in CpG detection within genomic features, indicating that the performance of RREM-seq in genomic element detection is not correlated with input concentration at these input levels. Both standard-input RREM-seq and low-input RREM-seq covered >85% of these regulatory genomic elements on average at 10× minimum coverage and showed superior performance compared with low-input WGEM-seq and RRBS (Supplementary Fig. S4A–D). Within GC-rich CpGI and promoter regions, RREM-seq (standard-input and low-input) on average generated ∼10 times more CpG calls (read count of each covered CpG) compared with low-input WGEM-seq and ∼4 times more CpG calls compared with RRBS (Fig. 2D and E). Overall, our results demonstrate that standard-input and low-input RREM-seq provide superior CpG detection with higher confidence in GC-rich genomic regions compared with RRBS and low-input WGEM-seq.

**Figure 3.**
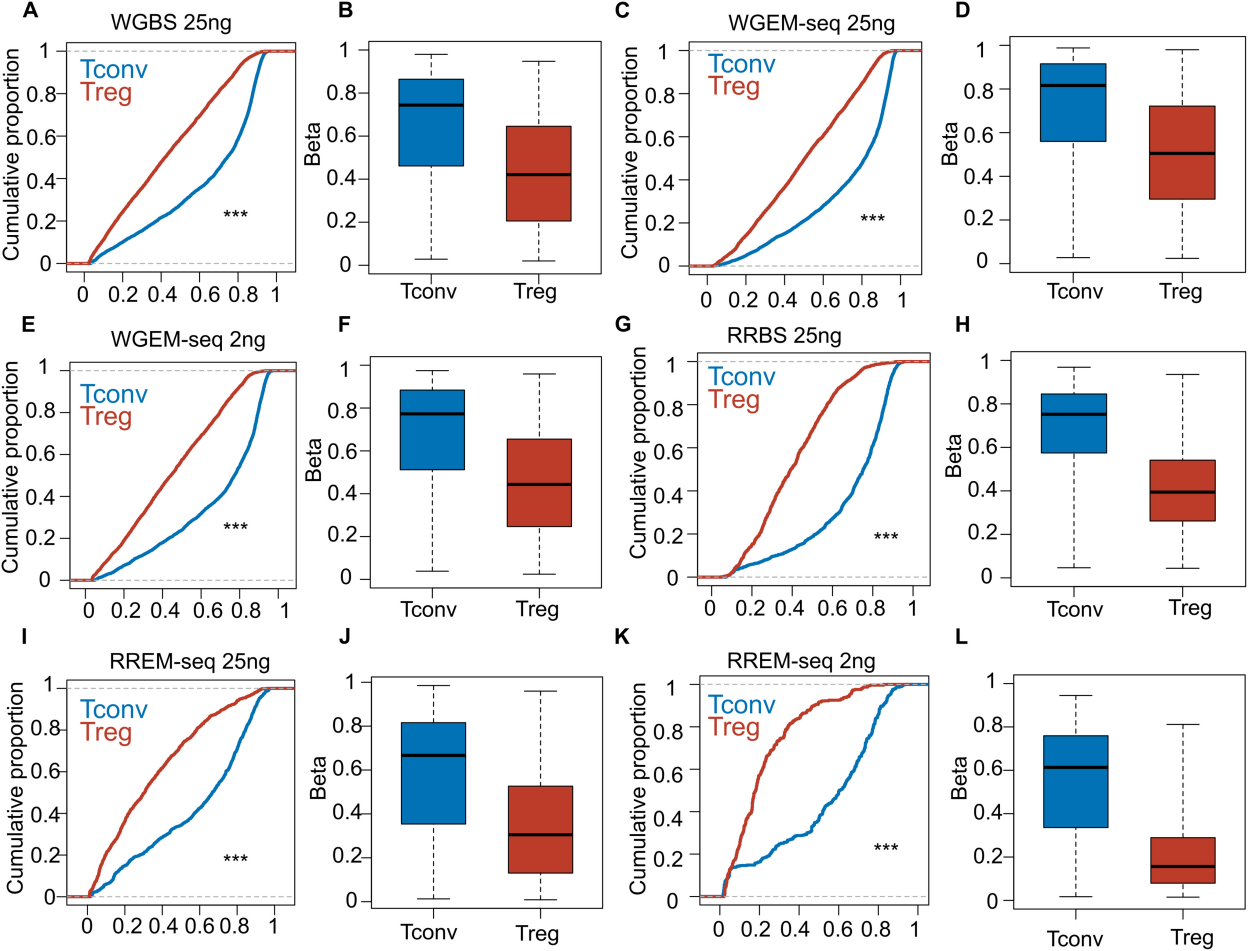
CpG methylation in Treg-SE elements. β Values of differentially methylated CpGs across Treg-SE in Tconv and Treg cells in WGBS (**A**and **B**; *n* = 21 367), WGEM-seq (standard input: **C** and **D**; *n* = 17 010; low input: **E** and **F**; *n* = 14 933), RRBS (**G** and **H**; *n* = 5037), and RREM-seq (standard input: **I** and **J**; *n* = 5469; low input: **K** and **L**; *n* = 1423) libraries, FDR *q* < 0.05. In panels (A), (C), (E), (G), (I), and (K), the cumulative distribution function of β values across the Treg-SE is plotted against their cumulative proportion. A leftward shift indicates hypomethylation. (B, D, F, H, J, L) Box-and-whisker plots comparing β values across the Treg-SE between Tconv and Treg cells. Data represent merged average of five biological replicates for each cell type. β values continuously range from 0 (unmethylated) to 1 (methylated). ***P < .0001 according to the Kolmogorov–Smirnov test.

Raw quantification of CpG methylation surrounding the transcriptional start site (TSS) also demonstrated the classic dip-and-plateau trend [17], indicating hypomethylation around the TSSs and hypermethylation within gene bodies (Supplementary Fig. S5A–F). We also observed average hypomethylation across CpGIs in all sequencing methods (Supplementary Fig. S5G–L). To validate the performance of RREM-seq in identifying methylation differences between Tconv and Treg cells, we quantified methylation levels within the Treg-SEs (Fig. 3A–L) [10]. In both standard-input and low-input RREM-seq libraries, CpG methylation across Treg-SEs demonstrated relative hypomethylation in Treg cells (Fig. 3I–L).

**Figure 4.**
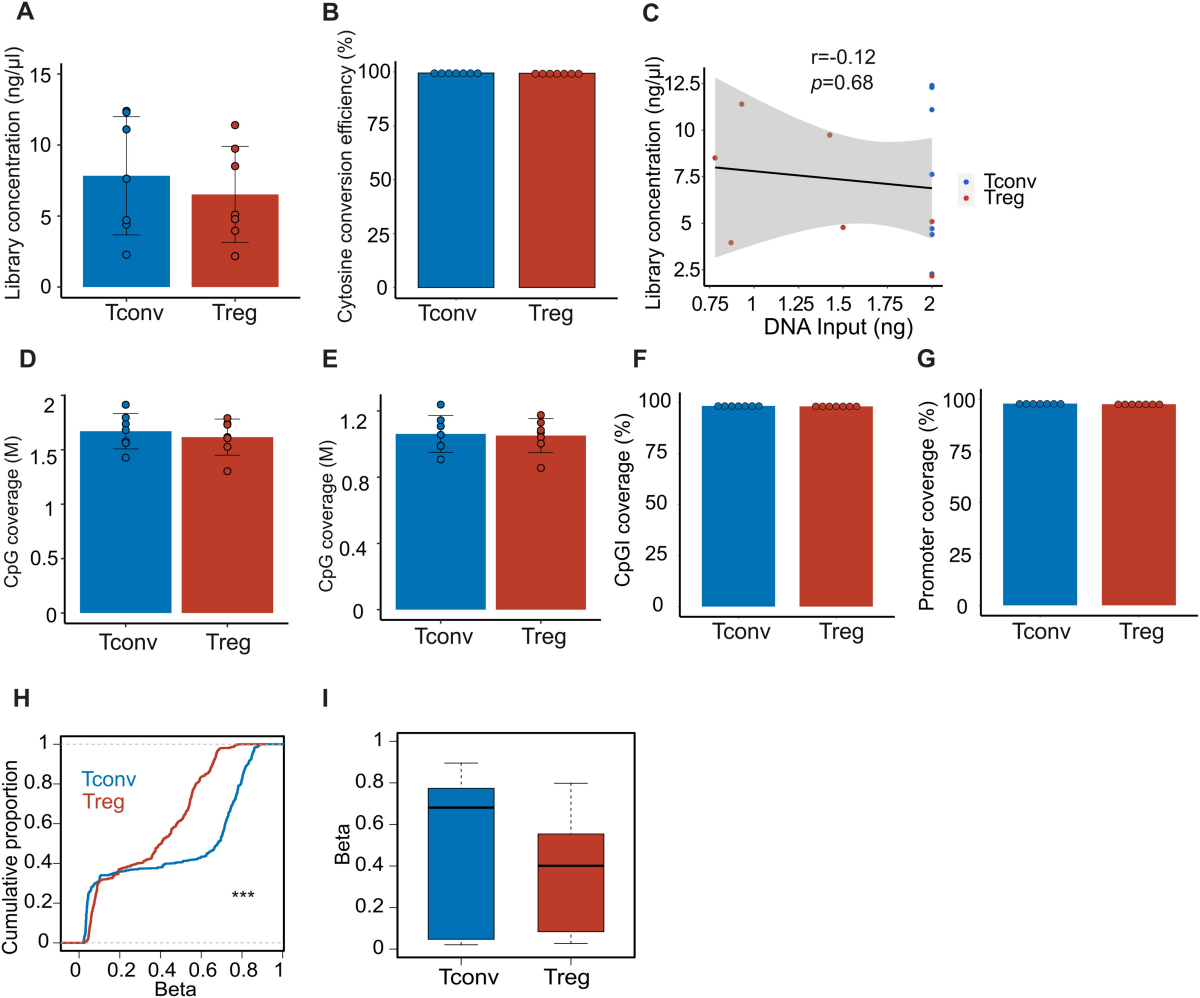
Methylation profiling of ultra-low-input alveolar T cell samples from patients with severe SARS-CoV-2 pneumonia. Final library concentration (**A**) and cytosine conversion efficiency (**B**) in alveolar Tconv and Treg cells. (**C**) Correlation plot comparing RREM-seq library concentration with sample DNA input. The black line and shaded area represent the linear regression fit line with 95% confidence interval. CpGs detected in CpGIs and the surrounding CpGI shores (**D**) and promoters (**E**) at 1× minimum threshold. Coverage of CpGIs and the surrounding CpGI shores (**F**) and promoters (**G**) at 1× minimum threshold. (**H**) Cumulative distribution function comparing β values of differentially methylated CpGs (*n* = 1759) within Treg-SE between alveolar Tconv and Treg cells, FDR *q* < 0.05. (**I**) β values of differentially methylated CpGs within Treg-SE. Data represent merged average of seven replicates for each cell type obtained from seven individual patients. ***P < .0001 by the Kolmogorov–Smirnov test. Summary plots show all data points with mean, and error bars show standard deviation.

The T cell response to lower respiratory tract infection is a critical determinant of outcomes in patients with severe pneumonia [18, 19]. To further demonstrate the performance of RREM-seq in assessing low-input clinical samples, we performed methylation profiling of alveolar T cells obtained by BAL from mechanically ventilated patients with severe SARS-CoV-2 pneumonia within 48 h of intubation. Alveolar T cells were sorted from BAL fluid samples that were collected as part of the SCRIPT study, an observational cohort study of mechanically ventilated patients with severe pneumonia [20]. As described in previous publications [9, 20], we used flow cytometry to isolate alveolar CD4^+^ CD127low CD25^+^ Treg cells and non-Treg CD4^+^ cells. We obtained seven paired Tconv (average cell count = 33 584) and Treg (average cell count = 2946) cell samples. Methylation libraries were generated using ≤2 ng genomic DNA (2 ng for Tconv cells and 0.9–2 ng for Treg cells) via our optimized RREM-seq procedure. On average, the final library concentration was 6.8 ng/μl (Fig. 4A). Average CpG methylation was 0.5% for unmethylated λ-bacteriophage DNA, indicating a >99% unmethylated cytosine conversion rate (Fig. 4B). We observed no significant correlation between sample DNA input and final library concentration (*r* = −0.12, *P*= .68) (Fig. 4C). These RREM-seq libraries also demonstrated similar coverage of CpGIs and promoters to standard-input RRBS libraries, confirming RREM-seq’s performance in profiling low-input clinical samples (Fig. 4D–G).

We previously demonstrated that the distinct Treg-SE DNA methylation patterns present in mouse Treg and Tconv cells are also found in Treg and Tconv cells in BAL fluid obtained from patients with severe pneumonia [9]. To compare methylation status within the Treg-SE regions in alveolar Tconv and Treg cells, we mapped 62 out of 66 regulatory elements from the original mm9 mouse reference genome onto the hg38 human reference genome. Alveolar Treg cells demonstrated significant hypomethylation across the Treg-SE regions compared with Tconv cells (Fig. 4H and I). These results in ultra-low-input samples are consistent with our previous publication [9], in which we used RRBS to compare methylation levels between alveolar Tconv cells and Treg cells across the Treg-SE regions in standard input samples. We also compared raw quantification of CpG methylation of Tconv and Treg cells in each patient; the Treg-SE DNA methylation pattern varied between patients (Supplementary Fig. S6).

## Discussion

Due to fewer sequencing reads required per sample, reduced representation methods offer a cost-effective alternative to whole-genome methylation sequencing approaches [4]. While RRBS and RREM-seq show comparable performance in global CpG detection at standard input levels, our results demonstrate that RREM-seq outperforms RRBS at ≤2-ng input levels, at which RRBS could not generate high-quality libraries. We also found that standard-input and low-input RREM-seq outperform RRBS in detecting CpGs within CpG-rich regulatory genomic elements at 5× and 10× minimum coverage, demonstrating RREM-seq’s superior performance in reduced representation methylation sequencing. Our results also demonstrated RREM-seq’s performance in profiling methylation status in ultra-low-input clinical samples, determining that RREM-seq detects Treg cell-specific hypomethylation patterns in alveolar Treg cells compared with alveolar Tconv cells in patients with severe SARS-CoV-2 pneumonia.

Bisulfite sequencing, the current gold standard, is limited by several important caveats. Bisulfite treatment involves deamination of cytosine residues in single-stranded DNA at 37–50°C followed by bisulfite removal using extreme pH adjustment (pH >13) [21]. These chemical reactions result in substantial DNA degradation, observed in various bisulfite conversion protocols [6, 21]. On top of this bisulfite-induced DNA degradation, the CpG enrichment step in reduced representation protocols has a yield of around 5%–10% of the original input after DNA fragment size selection, further lowering DNA input. Hence, the required DNA input for RRBS may not be feasible for many clinical samples and rare cell populations. Compared with bisulfite treatment, enzymatic conversion of cytosine residues does not contribute to DNA degradation, the conversion process does not require extreme pH, and the reaction temperature is kept at no higher than 37°C [5]. In addition to 5mC detection, the EM-seq pipeline can be modified to specifically identify 5hmC by incorporating the enzyme T4-GBT for 5hmC glycosylation.

Rubenstein and Solomon’s paper [22] developed another targeted methylation sequencing method (TEEM-seq) using enzyme-based cytosine conversion technology. While TEEM-seq used custom hybridization capture bait sets for enrichment, our RREM-seq method has the advantage of unbiased CpG enrichment: the restriction digestion step in our RREM-seq pipeline randomly enriches for CpG-rich regions and does not require additional hybridization bait design. Compared with methylation arrays, RREM-seq has the advantage of providing unbiased sampling and higher CpG coverage at similar cost. Illumina’s Infinium MethylationEPIC, one of the most used methylation array methods, requires around 200 ng genomic DNA and covers around 0.9 million CpGs in human samples and around 0.3 million CpGs in mouse samples. In contrast, our RREM-seq pipeline can reliably generate methylation libraries using <2-ng samples, has a similar cost (around $450 per sample at the time of publication) compared to MethylationEPIC, and provides 5× coverage of >1.5 million CpGs on average using low-input samples. With its unbiased CpG enrichment approach and comprehensive coverage, RREM-seq provides an opportunity to profile low-input samples at a relatively low cost.

Similar to the bisulfite-based methods, the accuracy of CpG detection heavily relies on the efficient conversion of unmethylated cytosine residues to uracil residues. Unconverted cytosine residues are categorized as 5mC by the downstream analysis, and therefore result in inaccurate methylation profiling. Our results confirmed that enzymatic conversion is highly efficient in converting unmethylated cytosines. Regardless, unmethylated negative control DNA (e.g. λ-bacteriophage) should be incorporated into the RREM-seq pipeline to address this potential limitation.

Overall, our results demonstrate that RREM-seq is a reliable method to perform methylation profiling and displays superior signal-to-noise ratios compared with RRBS. Many low-input samples have great potential to answer relevant research questions, yet the current RRBS pipeline cannot generate reliable methylation libraries using ultra-low input levels. Combining a reduced representation strategy with enzymatic conversion technology provides an opportunity to perform cost-effective methylation profiling of biologically relevant yet limited cell populations, especially in clinical samples.

## Supplementary Material

gkaf558_Supplemental_File

## Data Availability

Raw sequencing data of the mouse samples and processed CpG coverage, bedgraph, bigwig, and 1× SeqMonk probe files have been deposited to the GEO repository GSE266961. Raw sequencing data of the patient samples will be available via dbGaP under accession number phs002300.v1.p1. Custom functions for data analysis are available at GitHub (https://github.com/palupaca/RREMseq2024) or Figshare (https://doi.org/10.6084/m9.figshare.29212505). An example SeqMonk file was uploaded to Figshare (https://doi.org/10.6084/m9.figshare.28628168.v1).
