# Supplementary material for: Novel enzyme-based reduced representation method for DNA methylation profiling with low inputs
Source: Nucleic Acids Res. 2025 Jun 30;53(12):gkaf558. doi: 10.1093/nar/gkaf558 (PMC12207400; doi:10.1093/nar/gkaf558)
Supplement: gkaf558_Supplemental_File [file gkaf558_supplemental_file.pdf]

Supplemental materials

Novel enzyme-based reduced representation method for DNA methylation profiling with low inputs

Qianli Liu[1,2], Kathryn A. Helmin[1], Zachary D. Dortzbach[1], Carla P. Reyes Flores[1,2], Manuel A. Torres Acosta[1,3], Jonathan K. Gurkan[1,2,3], Anthony M. Joudi[1], Nurbek Mambetsariev[4], Luisa Morales-Nebreda[1], Mengjia Kang[1], Luke Rasmussen[5], Xóchitl G. Pérez-Leonor[1], Hiam Abdala-Valencia[1], and Benjamin D. Singer[1,6,7,8]

[1]Division of Pulmonary and Critical Care Medicine, Northwestern University Feinberg School of Medicine, Chicago, IL 60611 USA
[2]Driskill Graduate Program, Northwestern University Feinberg School of Medicine, Chicago, IL 60611 USA
[3]Medical Scientist Training Program, Northwestern University Feinberg School of Medicine, Chicago, IL 60611 USA
[4]Division of Allergy and Immunology, Northwestern University Feinberg School of Medicine, Chicago, IL 60611 USA
[5]Division of Health and Biomedical Informatics, Department of Preventive Medicine, Northwestern University Feinberg School of Medicine, Chicago, IL 60611 USA
[6]Department of Biochemistry and Molecular Genetics, Northwestern University Feinberg School of Medicine, Chicago, IL 60611 USA
[7]Simpson Querrey Institute for Epigenetics, Northwestern University Feinberg School of Medicine, Chicago, IL 60611 USA
[8]Simpson Querrey Lung Institute for Translational Science (SQ LIFTS), Northwestern University Feinberg School of Medicine, Chicago, IL 60611 USA.

To whom correspondence should be addressed:
Benjamin D. Singer, MD
Division of Pulmonary and Critical Care Medicine, Department of Medicine
Department of Biochemistry and Molecular Genetics
Northwestern University Feinberg School of Medicine
303 E. Superior St., Simpson Querrey 5th Floor
Chicago, IL 60611 USA
benjamin-singer@northwestern.edu
Tel: (312) 503-4494
Fax: (312) 503-0411

Reagent/Antigen	Conjugate	Clone	Company	Catalog no.
CD3ε	PE	145-2C11	eBioscience	12-0031-83
CD4	PerCP-Cy5.5	GK1.5	Biolegend	100434
CD25	APC	PC61.5	Invitrogen	2154040
Fixable Viability Dye eFluor 506	N/A	N/A	eBioscience	65-0866-14

Supplemental Table 1. Flow cytometry fluorochromes used for sorting mouse T cells.

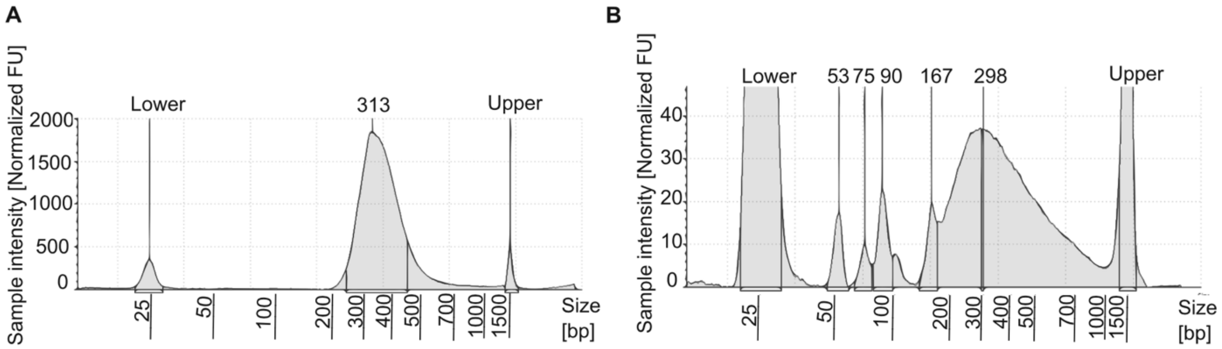

Supplemental Figure 1. **TapeStation traces of low-input DNA methylation libraries.** (**A-B**) Final library quality of a representative RREM-seq library generated using 1-ng input (A) and a representative RRBS library using 2-ng input (B). bp, base pair; FU, fluorescence units.

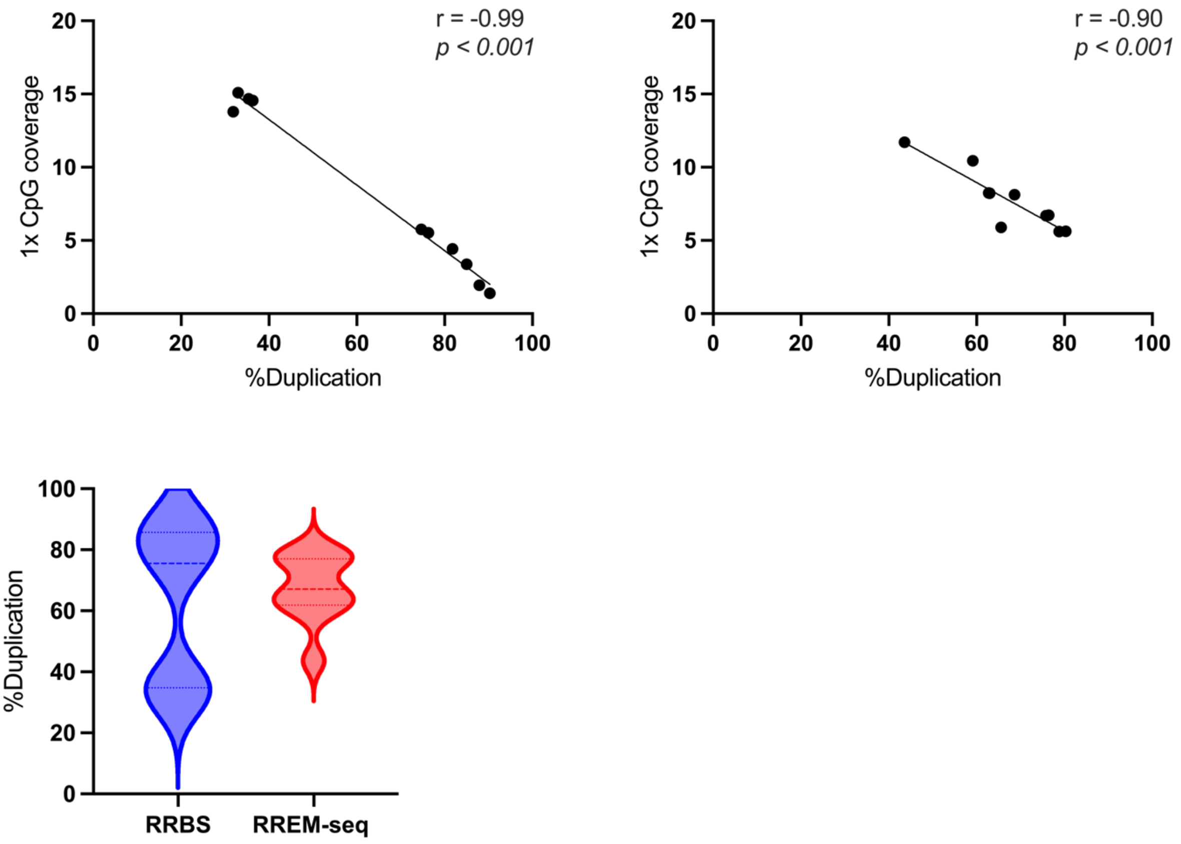

Supplemental Figure 2. Library duplication in RRBS and RREM-seq. (**A-B**) Correlation plot comparing 1x CpG coverage with library duplication rate in standard-input RRBS (A) and RREM-seq (B). (**C**) Violin plot comparing duplication rate between standard-input RRBS and RREM-seq.

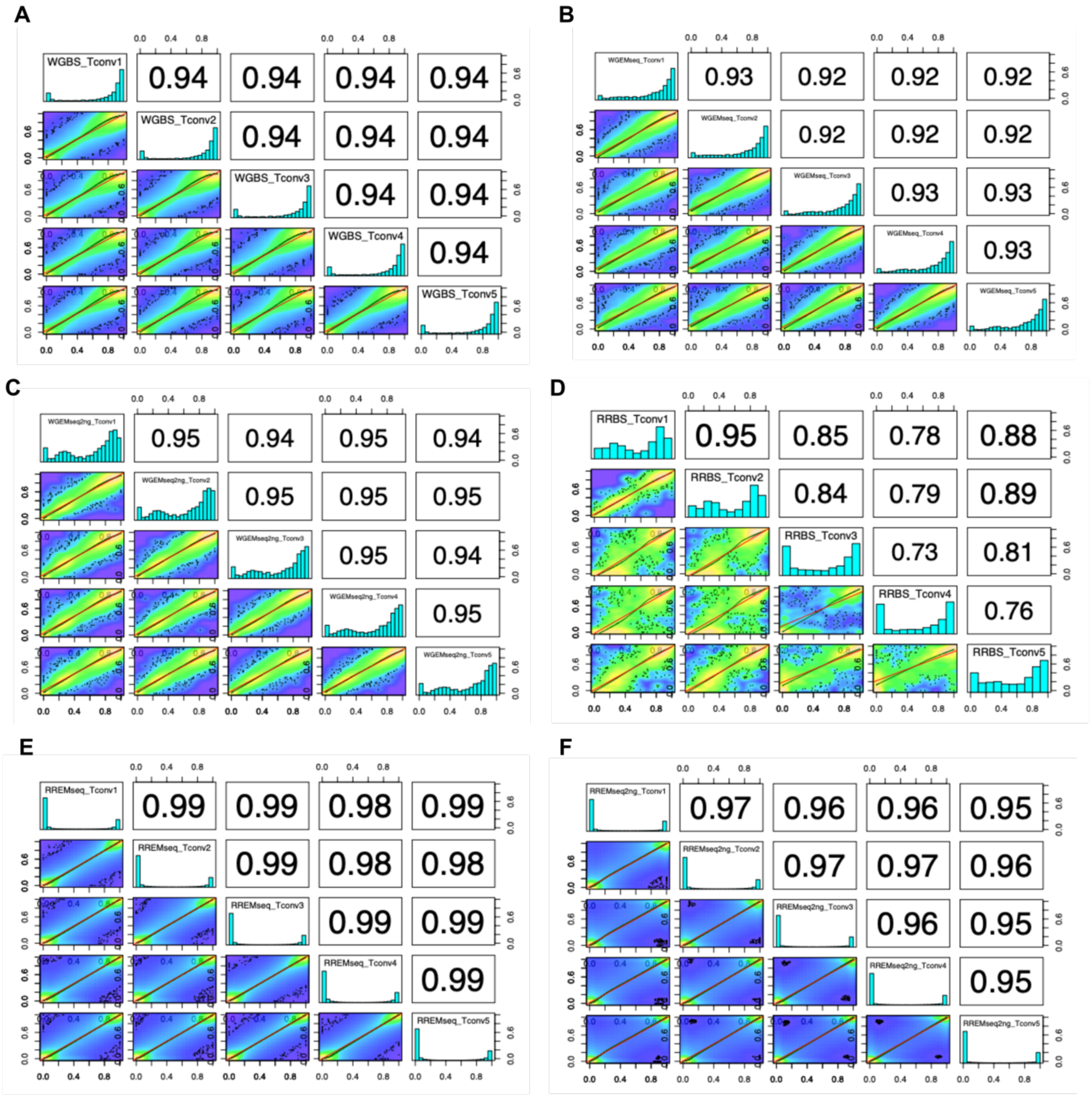
A
WGBS_Tconv1
WGBS_Tconv2
WGBS_Tconv3
WGBS_Tconv4
WGBS_Tconv5
0.94 0.94 0.94 0.94
0.94 0.94 0.94
0.94 0.94
0.94
B
WGEMseq_Tconv1
WGEMseq_Tconv2
WGEMseq_Tconv3
WGEMseq_Tconv4
WGEMseq_Tconv5
0.93 0.92 0.92 0.92
0.92 0.92 0.92
0.93 0.93
0.93
C
WGEMseq2ng_Tconv1
WGEMseq2ng_Tconv2
WGEMseq2ng_Tconv3
WGEMseq2ng_Tconv4
WGEMseq2ng_Tconv5
0.95 0.94 0.95 0.94
0.95 0.95 0.95
0.95 0.94
0.95
D
RRBS_Tconv1
RRBS_Tconv2
RRBS_Tconv3
RRBS_Tconv4
RRBS_Tconv5
0.95 0.85 0.78 0.88
0.84 0.79 0.89
0.73 0.81
0.76
E
RREMseq_Tconv1
RREMseq_Tconv2
RREMseq_Tconv3
RREMseq_Tconv4
RREMseq_Tconv5
0.99 0.99 0.98 0.99
0.99 0.98 0.98
0.99 0.99
0.99
F
RREMseq2ng_Tconv1
RREMseq2ng_Tconv2
RREMseq2ng_Tconv3
RREMseq2ng_Tconv4
RREMseq2ng_Tconv5
0.97 0.96 0.96 0.95
0.97 0.97 0.96
0.96 0.95
0.95

G

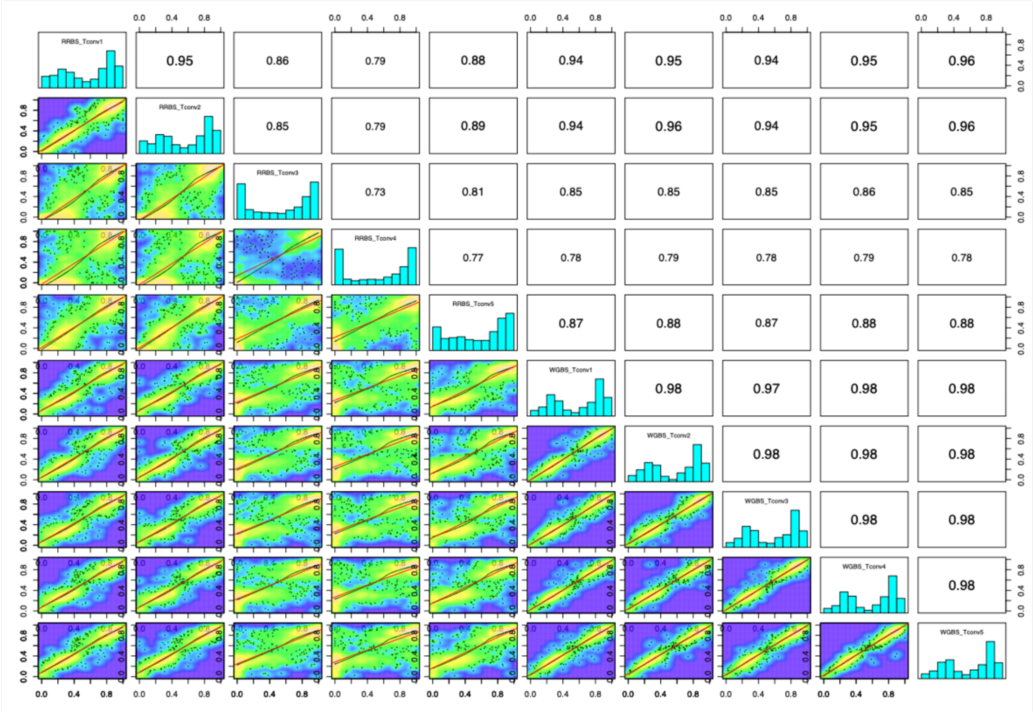
RRBS_Tconv1
RRBS_Tconv2
RRBS_Tconv3
RRBS_Tconv4
RRBS_Tconv5
WGBS_Tconv1
WGBS_Tconv2
WGBS_Tconv3
WGBS_Tconv4
WGBS_Tconv5
0.95 0.86 0.79 0.88 0.94 0.95 0.94 0.95 0.96
0.85 0.79 0.89 0.94 0.96 0.94 0.95 0.96
0.73 0.81 0.85 0.85 0.85 0.86 0.85
0.77 0.78 0.79 0.78 0.79 0.78
0.87 0.88 0.87 0.88 0.88
0.98 0.97 0.98 0.98
0.98 0.98 0.98
0.98 0.98
0.98
0.0 0.4 0.8

H

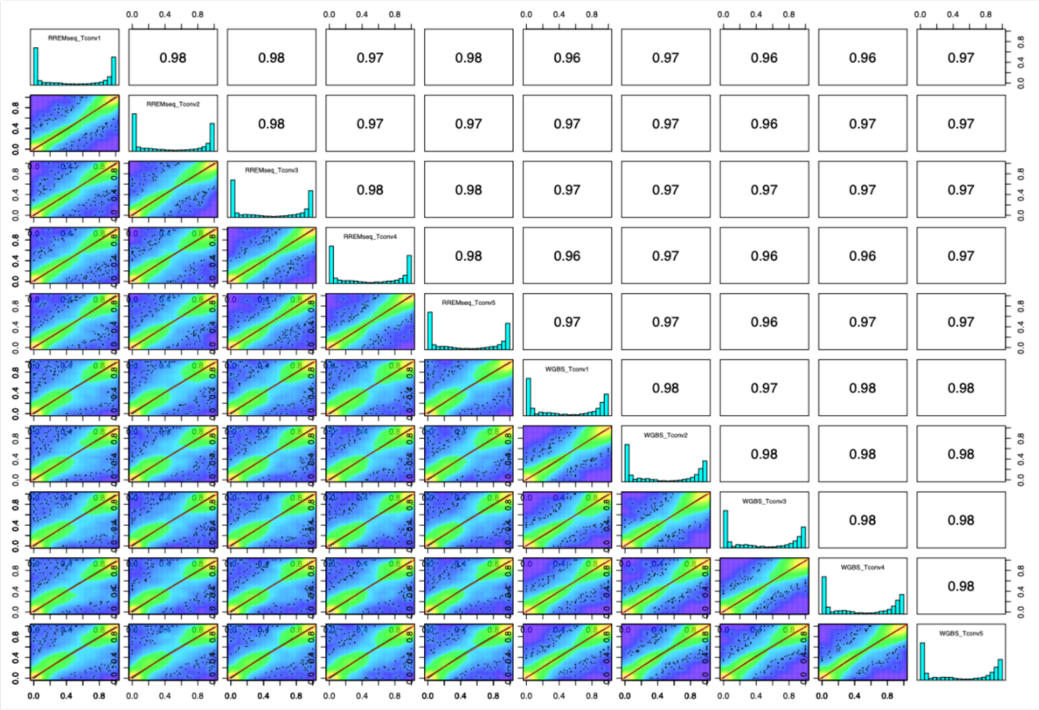
RREMseq_Tconv1
RREMseq_Tconv2
RREMseq_Tconv3
RREMseq_Tconv4
RREMseq_Tconv5
WGBS_Tconv1
WGBS_Tconv2
WGBS_Tconv3
WGBS_Tconv4
WGBS_Tconv5
0.98 0.98 0.97 0.98 0.96 0.97 0.96 0.96 0.97
0.98 0.97 0.97 0.97 0.97 0.96 0.97 0.97
0.98 0.98 0.97 0.97 0.97 0.97 0.97
0.98 0.96 0.97 0.96 0.96 0.97
0.97 0.97 0.96 0.97 0.97
0.98 0.97 0.98 0.98
0.98 0.98 0.98
0.98 0.98
0.98
0.0 0.4 0.8

I

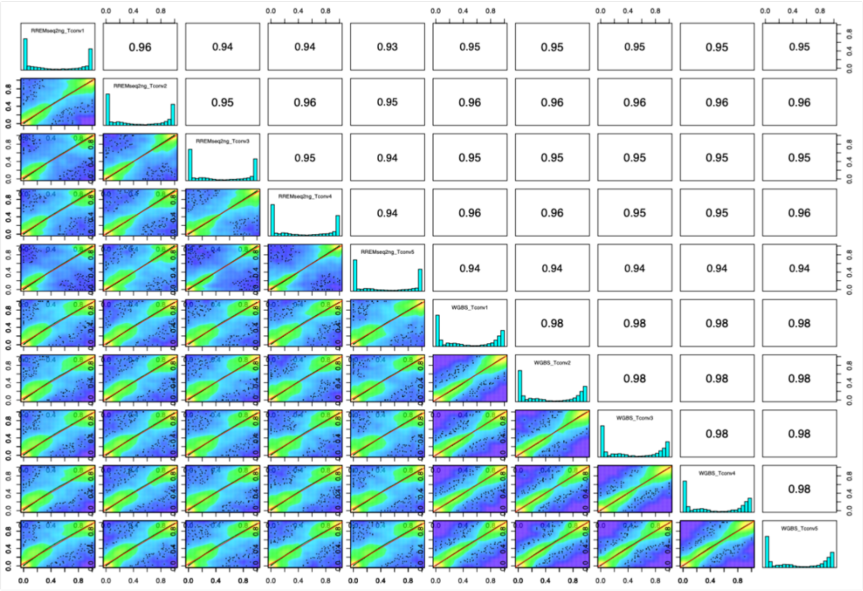
0.96 0.94 0.94 0.93 0.95 0.95 0.95 0.95 0.95
0.95 0.96 0.95 0.96 0.96 0.96 0.96 0.96
0.95 0.94 0.95 0.95 0.95 0.95 0.95
0.94 0.96 0.96 0.95 0.95 0.96
0.94 0.94 0.94 0.94 0.94
0.98 0.98 0.98 0.98
0.98 0.98 0.98
0.98 0.98
0.98
0.0 0.4 0.8

J

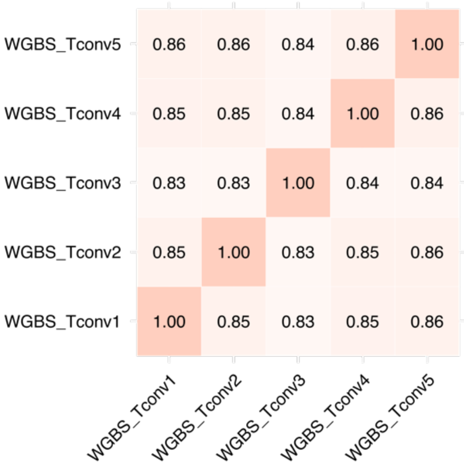
WGBS_Tconv5 0.86 0.86 0.84 0.86 1.00
WGBS_Tconv4 0.85 0.85 0.84 1.00 0.86
WGBS_Tconv3 0.83 0.83 1.00 0.84 0.84
WGBS_Tconv2 0.85 1.00 0.83 0.85 0.86
WGBS_Tconv1 1.00 0.85 0.83 0.85 0.86
WGBS_Tconv1
WGBS_Tconv2
WGBS_Tconv3
WGBS_Tconv4
WGBS_Tconv5

K

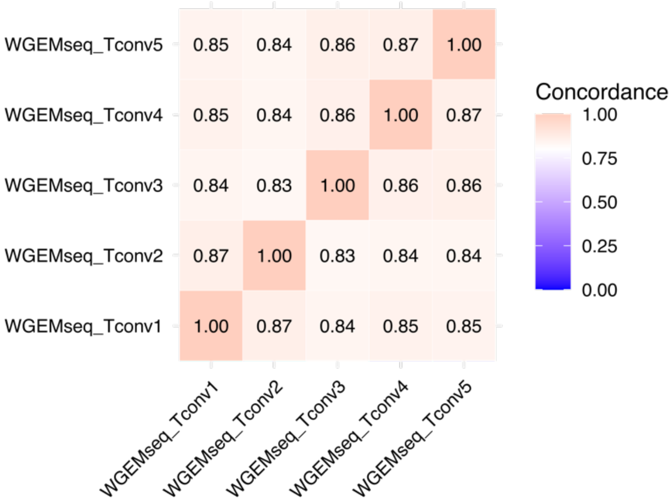
WGEMseq_Tconv5 0.85 0.84 0.86 0.87 1.00
WGEMseq_Tconv4 0.85 0.84 0.86 1.00 0.87
WGEMseq_Tconv3 0.84 0.83 1.00 0.86 0.86
WGEMseq_Tconv2 0.87 1.00 0.83 0.84 0.84
WGEMseq_Tconv1 1.00 0.87 0.84 0.85 0.85
WGEMseq_Tconv1
WGEMseq_Tconv2
WGEMseq_Tconv3
WGEMseq_Tconv4
WGEMseq_Tconv5
Concordance
1.00
0.75
0.50
0.25
0.00

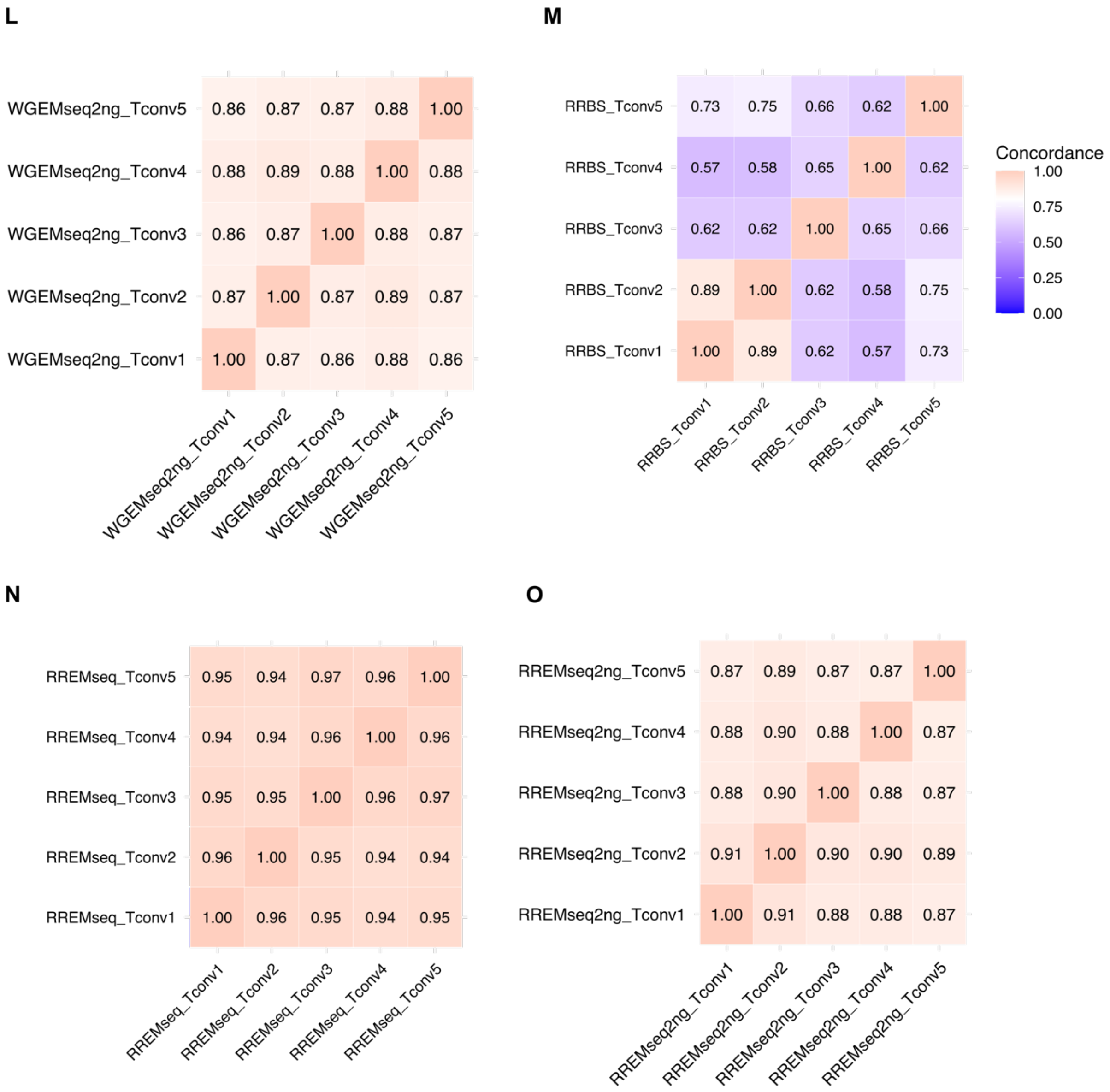
L
WGEMseq2ng_Tconv5 0.86 0.87 0.87 0.88 1.00
WGEMseq2ng_Tconv4 0.88 0.89 0.88 1.00 0.88
WGEMseq2ng_Tconv3 0.86 0.87 1.00 0.88 0.87
WGEMseq2ng_Tconv2 0.87 1.00 0.87 0.89 0.87
WGEMseq2ng_Tconv1 1.00 0.87 0.86 0.88 0.86
WGEMseq2ng_Tconv1 WGEMseq2ng_Tconv2 WGEMseq2ng_Tconv3 WGEMseq2ng_Tconv4 WGEMseq2ng_Tconv5
M
RRBS_Tconv5 0.73 0.75 0.66 0.62 1.00
RRBS_Tconv4 0.57 0.58 0.65 1.00 0.62
RRBS_Tconv3 0.62 0.62 1.00 0.65 0.66
RRBS_Tconv2 0.89 1.00 0.62 0.58 0.75
RRBS_Tconv1 1.00 0.89 0.62 0.57 0.73
RRBS_Tconv1 RRBS_Tconv2 RRBS_Tconv3 RRBS_Tconv4 RRBS_Tconv5
Concordance
1.00
0.75
0.50
0.25
0.00
N
RREMseq_Tconv5 0.95 0.94 0.97 0.96 1.00
RREMseq_Tconv4 0.94 0.94 0.96 1.00 0.96
RREMseq_Tconv3 0.95 0.95 1.00 0.96 0.97
RREMseq_Tconv2 0.96 1.00 0.95 0.94 0.94
RREMseq_Tconv1 1.00 0.96 0.95 0.94 0.95
RREMseq_Tconv1 RREMseq_Tconv2 RREMseq_Tconv3 RREMseq_Tconv4 RREMseq_Tconv5
O
RREMseq2ng_Tconv5 0.87 0.89 0.87 0.87 1.00
RREMseq2ng_Tconv4 0.88 0.90 0.88 1.00 0.87
RREMseq2ng_Tconv3 0.88 0.90 1.00 0.88 0.87
RREMseq2ng_Tconv2 0.91 1.00 0.90 0.90 0.89
RREMseq2ng_Tconv1 1.00 0.91 0.88 0.88 0.87
RREMseq2ng_Tconv1 RREMseq2ng_Tconv2 RREMseq2ng_Tconv3 RREMseq2ng_Tconv4 RREMseq2ng_Tconv5

P

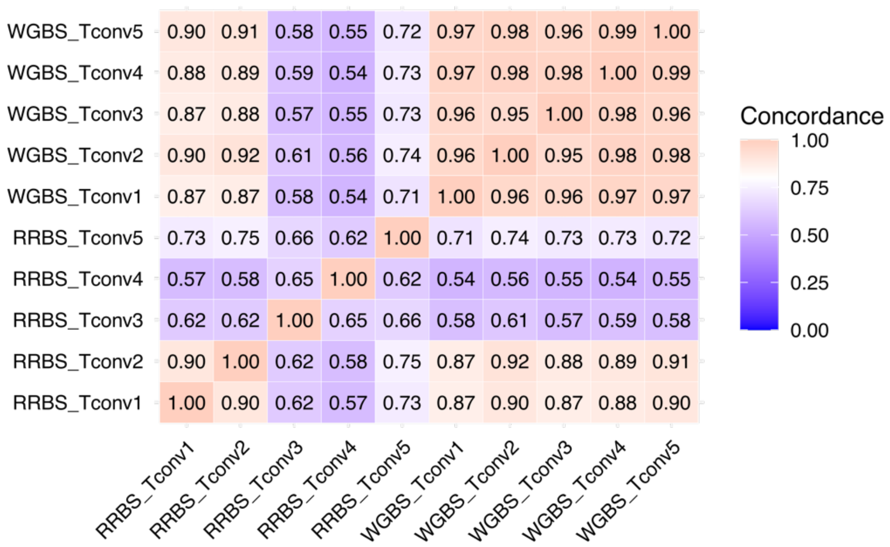
WGBS_Tconv5 0.90 0.91 0.58 0.55 0.72 0.97 0.98 0.96 0.99 1.00
WGBS_Tconv4 0.88 0.89 0.59 0.54 0.73 0.97 0.98 0.98 1.00 0.99
WGBS_Tconv3 0.87 0.88 0.57 0.55 0.73 0.96 0.95 1.00 0.98 0.96
WGBS_Tconv2 0.90 0.92 0.61 0.56 0.74 0.96 1.00 0.95 0.98 0.98
WGBS_Tconv1 0.87 0.87 0.58 0.54 0.71 1.00 0.96 0.96 0.97 0.97
RRBS_Tconv5 0.73 0.75 0.66 0.62 1.00 0.71 0.74 0.73 0.73 0.72
RRBS_Tconv4 0.57 0.58 0.65 1.00 0.62 0.54 0.56 0.55 0.54 0.55
RRBS_Tconv3 0.62 0.62 1.00 0.65 0.66 0.58 0.61 0.57 0.59 0.58
RRBS_Tconv2 0.90 1.00 0.62 0.58 0.75 0.87 0.92 0.88 0.89 0.91
RRBS_Tconv1 1.00 0.90 0.62 0.57 0.73 0.87 0.90 0.87 0.88 0.90
RRBS_Tconv1 RRBS_Tconv2 RRBS_Tconv3 RRBS_Tconv4 RRBS_Tconv5 WGBS_Tconv1 WGBS_Tconv2 WGBS_Tconv3 WGBS_Tconv4 WGBS_Tconv5
Concordance
1.00
0.75
0.50
0.25
0.00

Q

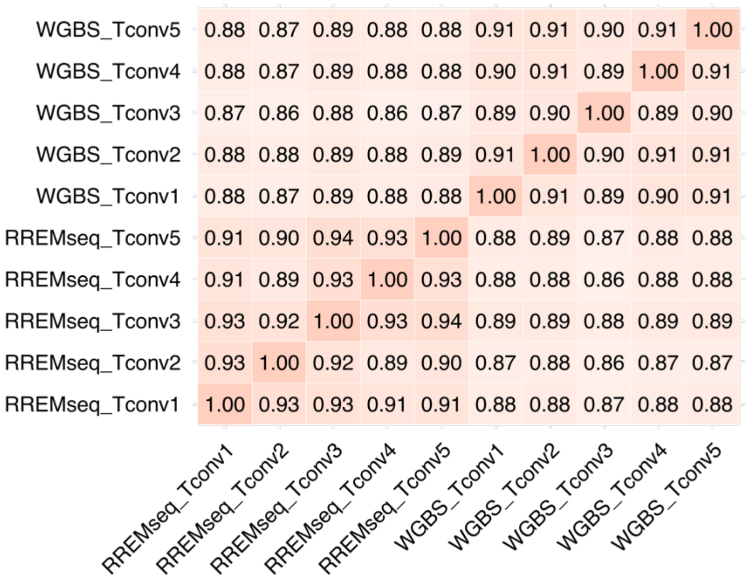
WGBS_Tconv5 0.88 0.87 0.89 0.88 0.88 0.91 0.91 0.90 0.91 1.00
WGBS_Tconv4 0.88 0.87 0.89 0.88 0.88 0.90 0.91 0.89 1.00 0.91
WGBS_Tconv3 0.87 0.86 0.88 0.86 0.87 0.89 0.90 1.00 0.89 0.90
WGBS_Tconv2 0.88 0.88 0.89 0.88 0.89 0.91 1.00 0.90 0.91 0.91
WGBS_Tconv1 0.88 0.87 0.89 0.88 0.88 1.00 0.91 0.89 0.90 0.91
RREMseq_Tconv5 0.91 0.90 0.94 0.93 1.00 0.88 0.89 0.87 0.88 0.88
RREMseq_Tconv4 0.91 0.89 0.93 1.00 0.93 0.88 0.88 0.86 0.88 0.88
RREMseq_Tconv3 0.93 0.92 1.00 0.93 0.94 0.89 0.89 0.88 0.89 0.89
RREMseq_Tconv2 0.93 1.00 0.92 0.89 0.90 0.87 0.88 0.86 0.87 0.87
RREMseq_Tconv1 1.00 0.93 0.93 0.91 0.91 0.88 0.88 0.87 0.88 0.88
RREMseq_Tconv1 RREMseq_Tconv2 RREMseq_Tconv3 RREMseq_Tconv4 RREMseq_Tconv5 WGBS_Tconv1 WGBS_Tconv2 WGBS_Tconv3 WGBS_Tconv4 WGBS_Tconv5

R

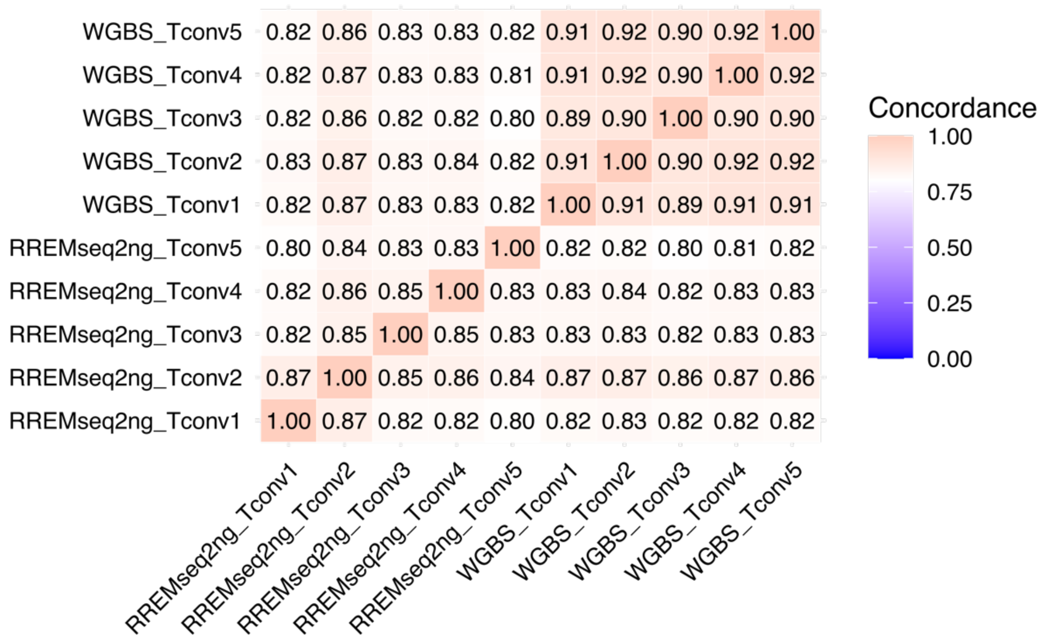

Supplemental Figure 3 Pairwise correlation and concordance analysis between methylation libraries. (**A-F**) Within-group correlations between replicates in WGBS (A, total number of CpG used for correlation analysis = 156,294), standard-input WGEM-seq (B, total number of CpG used for correlation analysis = 30,102), low-input WGEM-seq (C, total number of CpG used for correlation analysis = 13,224), RRBS (D, total number of CpG used for correlation analysis = 1,573), standard-input RREM-seq (E, total number of CpG used for correlation analysis = 984,588), and low-input RREM-seq (F, total number of CpG used for correlation analysis = 628,255). (**G-I**) Correlation analysis between WGBS and RRBS (G, total number of CpG used for correlation analysis = 1,481), between WGBS and standard-input RREM-seq (H, total number of CpG used for correlation analysis =14,136), and between WGBS and low-input RREM-seq (I, total number of CpG used for correlation analysis = 9,225). (**J-O**) Within-group methylation concordance analysis between replicates in WGBS (J), standard-input WGEM-seq (K), low-input WGEM-seq (L), RRBS (M), standard-input RREM-seq (N), and low-input RREM-seq (O). (**P-R**) Methylation concordance analysis between WGBS and RRBS (P), between WGBS and standard-input RREM-seq (Q), and between WGBS and low-input RREM-seq (R). In A-I, x-axis and y-axis of correlation plots represent beta values and numbers in the upper panel represent Pearson correlation coefficient from pairwise comparisons. In J-R, numbers in concordance matrix represent average pairwise concordant score (for each CpG site, a concordant call between two samples was defined as having an absolute difference in beta values of less than 0.15). Only CpGs detected at 10x minimum coverage in all replicates were included in each comparison.

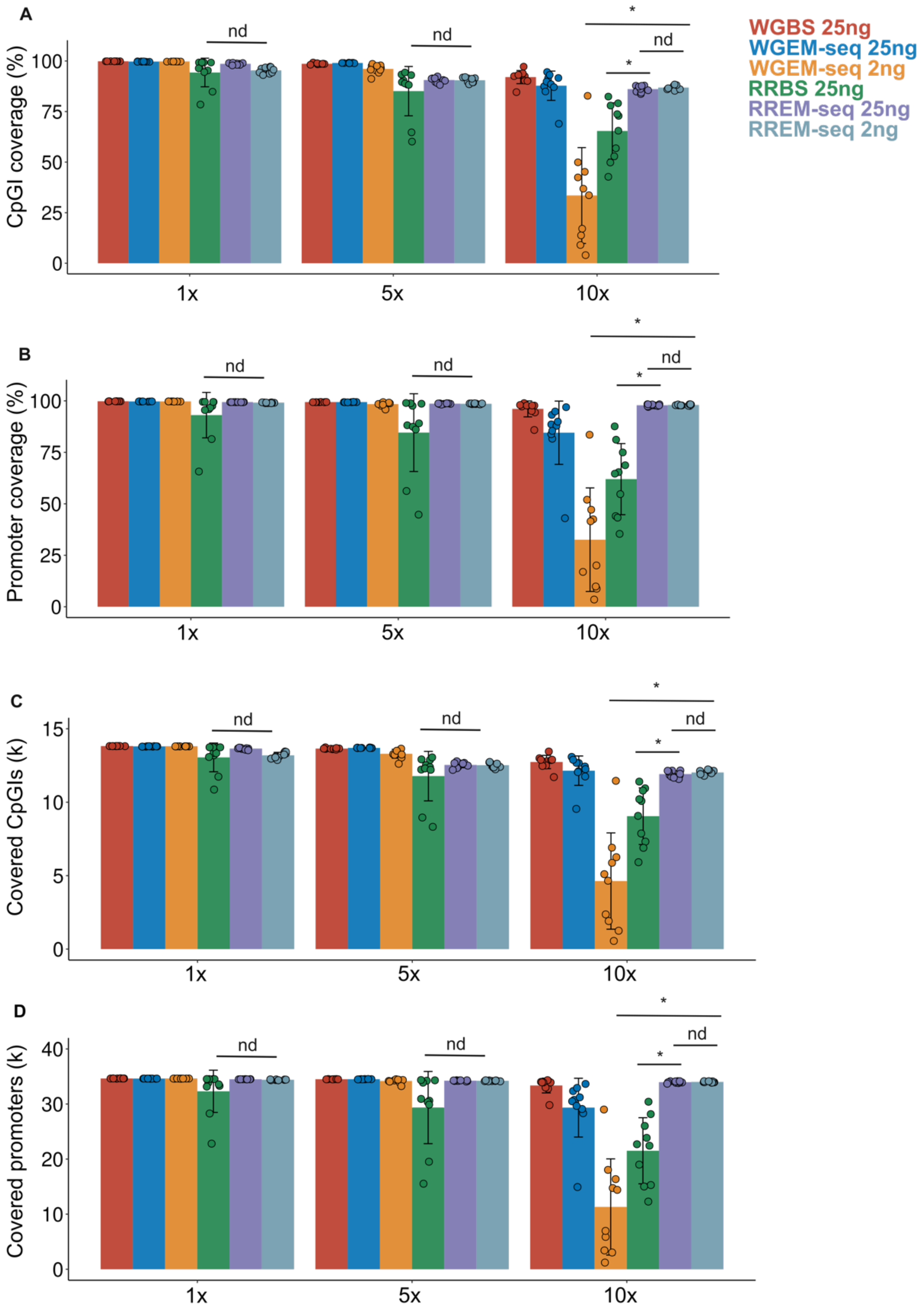
A
CpGI coverage (%)
B
Promoter coverage (%)
C
Covered CpGIs (k)
D
Covered promoters (k)
1x
5x
10x
nd
*
WGBS 25ng
WGEM-seq 25ng
WGEM-seq 2ng
RRBS 25ng
RREM-seq 25ng
RREM-seq 2ng

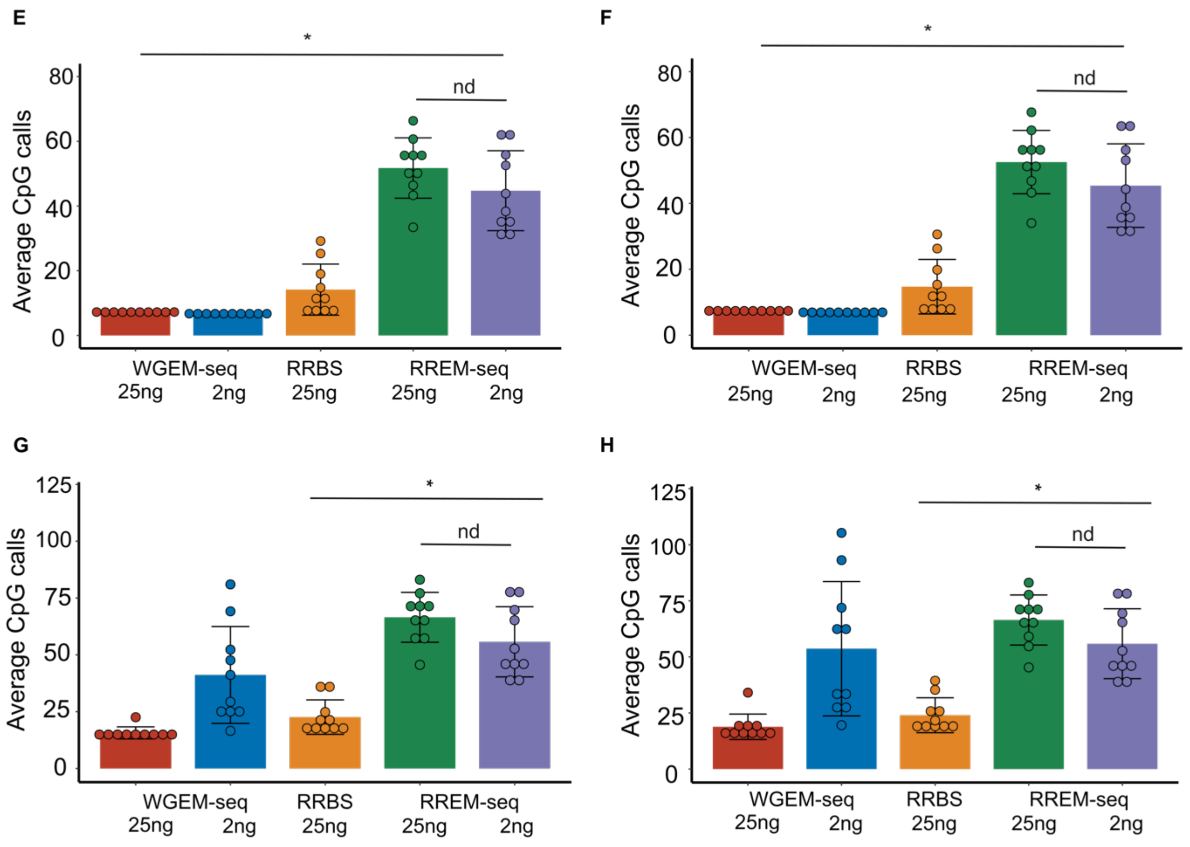

Supplemental Figure 4. Coverage of regulatory genomic elements. (**A-D**) CpGI (including the surrounding CpG island shores) coverage in percentage (A, out of 13,840 total CpGIs in the SeqMonk GRCm38 annotation set) and absolute count (C) at 1x, 5x, and 10x minimum thresholds (CpGs with at least 1, 5, and 10 reads were included in each analysis). Promoter coverage in percentage (B, out of 34,714 total promoters in the SeqMonk GRCm38 annotation set) and absolute count (D) at 1x, 5x, and 10x minimum thresholds (CpGs with at least 1, 5, and 10 reads were included in each analysis). (**E-H**) Average number of CpG calls (read count of a given CpG) within CpGIs, including the surrounding CpG island shores, (E,G) and promoters (F,H) at 5x and 10x minimum coverage (CpGs with at least 5 and 10 reads were included in each analysis). Analysis only includes CpGs with non-zero counts in each sample. Each data point represents one sample, and the bars represent average values of five biological replicates (5 Tconv cell samples and 5 Treg cell samples). Error bars show standard deviation. * $q < 0.05$; nd, not a discovery, according to one-way ANOVA (Kruskal-Wallis test) using the two-stage linear step-up procedure of Benjamini, Krieger, and Yekutieli with Q = 5%.

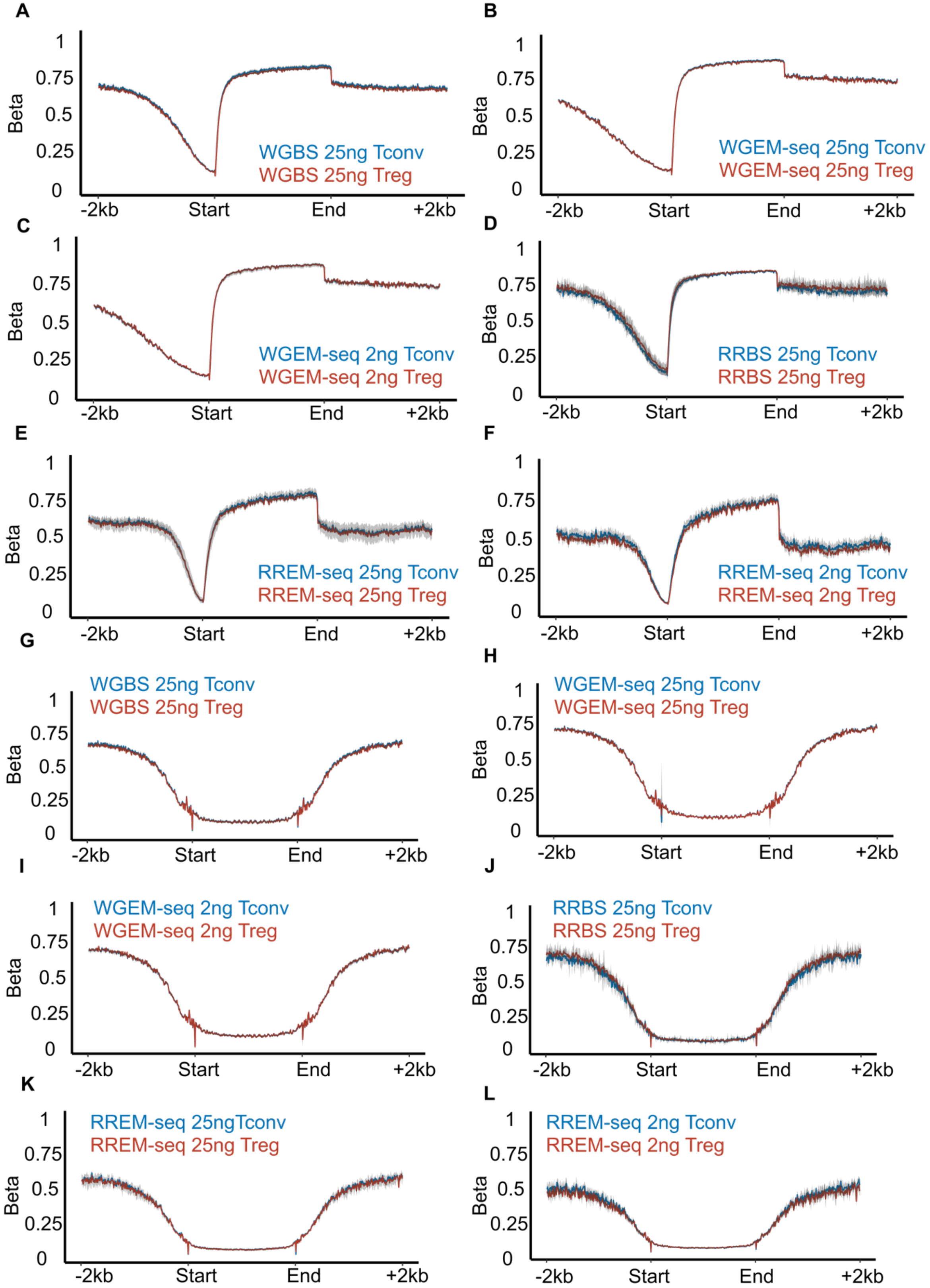
A
Beta
1
0.75
0.5
0.25
0
-2kb
Start
End
+2kb
WGBS 25ng Tconv
WGBS 25ng Treg
B
WGEM-seq 25ng Tconv
WGEM-seq 25ng Treg
C
WGEM-seq 2ng Tconv
WGEM-seq 2ng Treg
D
RRBS 25ng Tconv
RRBS 25ng Treg
E
RREM-seq 25ng Tconv
RREM-seq 25ng Treg
F
RREM-seq 2ng Tconv
RREM-seq 2ng Treg
G
WGBS 25ng Tconv
WGBS 25ng Treg
H
WGEM-seq 25ng Tconv
WGEM-seq 25ng Treg
I
WGEM-seq 2ng Tconv
WGEM-seq 2ng Treg
J
RRBS 25ng Tconv
RRBS 25ng Treg
K
RREM-seq 25ngTconv
RREM-seq 25ng Treg
L
RREM-seq 2ng Tconv
RREM-seq 2ng Treg

Supplemental Figure 5. CpG methylation in regulatory genomic elements. (**A-F**) SeqMonk quantification trend plots of β scores across gene bodies defined by transcriptional start site (TSS) and transcriptional end site (TES) with 2 kb of flanking sequence. (**G-L**) SeqMonk quantification trend plots of β scores across CpGIs with 2 kb of flanking sequence. Data represent merged average of 5 biological replicates for each cell type, and the shaded area represents the range.

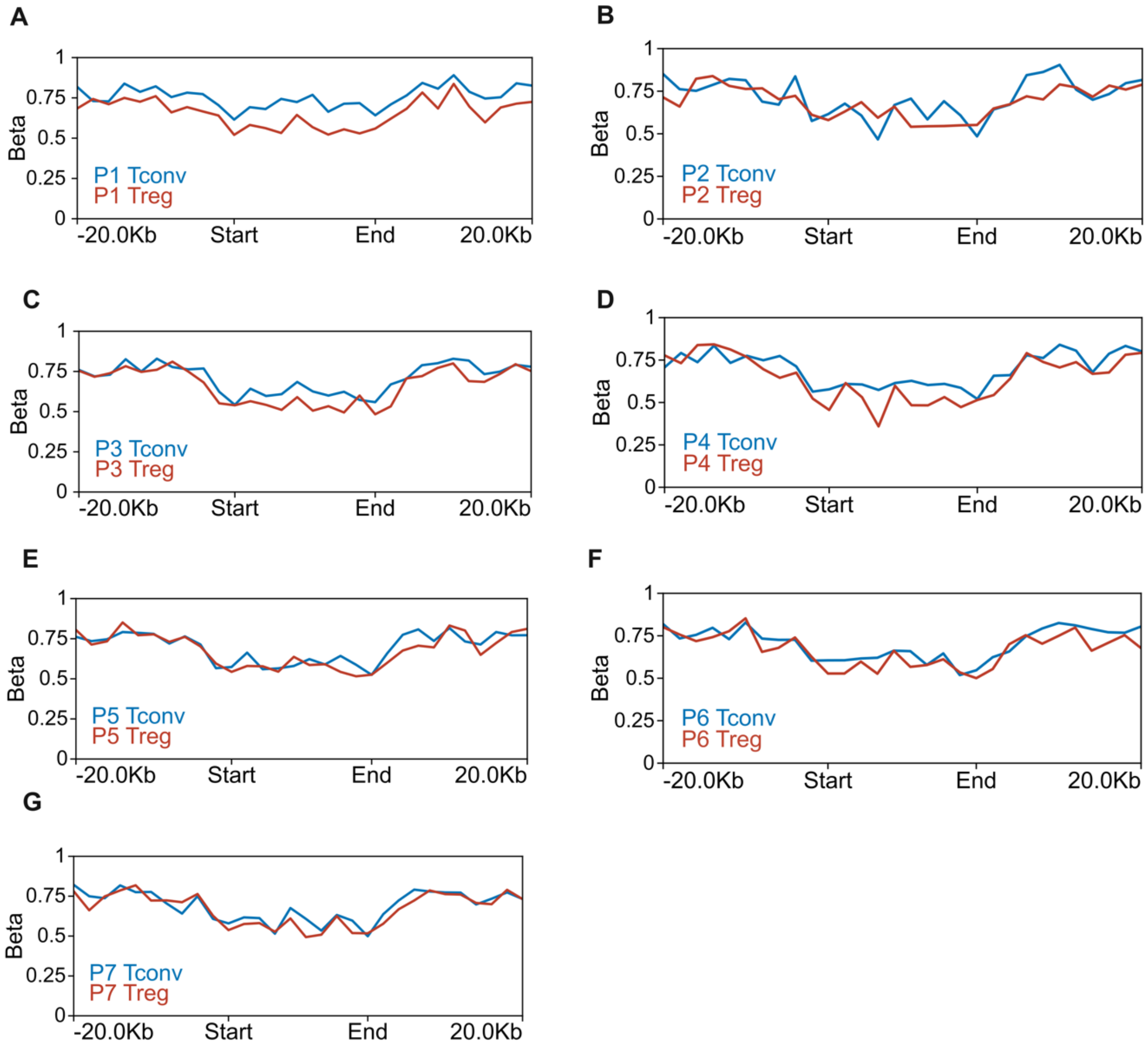

Supplemental Figure 6. Analysis of Treg-specific super-enhancer (Treg-SE) elements in patients with severe SARS-CoV-2 pneumonia. (**A-G**) SeqMonk quantification trend plot of raw DNA methylation (β scores) across Treg-SE with 20 kb of flanking sequence on either side from each patient.
